# Validation of functional calibration and strap-down joint drift correction for computing 3D joint angles of knee, hip, and trunk in alpine skiing

**DOI:** 10.1371/journal.pone.0181446

**Published:** 2017-07-26

**Authors:** Benedikt Fasel, Jörg Spörri, Pascal Schütz, Silvio Lorenzetti, Kamiar Aminian

**Affiliations:** 1 Laboratory of Movement Analysis and Measurement, Ecole Polytechnique Fédérale de Lausanne, Lausanne, Switzerland; 2 Department of Sport Science and Kinesiology, University of Salzburg, Hallein-Rif, Austria; 3 Department of Orthopaedics, Balgrist University Hospital, University of Zurich, Zurich, Switzerland; 4 Institute for Biomechanics, Eidgenössische Technische Hochschule Zurich, Zurich Switzerland; Universite de Nantes, FRANCE

## Abstract

To obtain valid 3D joint angles with inertial sensors careful sensor-to-segment calibration (i.e. functional or anatomical calibration) is required and measured angular velocity at each sensor needs to be integrated to obtain segment and joint orientation (i.e. joint angles). Existing functional and anatomical calibration procedures were optimized for gait analysis and calibration movements were impractical to perform in outdoor settings. Thus, the aims of this study were 1) to propose and validate a set of calibration movements that were optimized for alpine skiing and could be performed outdoors and 2) to validate the 3D joint angles of the knee, hip, and trunk during alpine skiing. The proposed functional calibration movements consisted of squats, trunk rotations, hip ad/abductions, and upright standing. The joint drift correction previously proposed for alpine ski racing was improved by adding a second step to reduce separately azimuth drift. The system was validated indoors on a skiing carpet at the maximum belt speed of 21 km/h and for measurement durations of 120 seconds. Calibration repeatability was on average <2.7° (i.e. 3D joint angles changed on average <2.7° for two repeated sets of calibration movements) and all movements could be executed wearing ski-boots. Joint angle precision was <4.9° for all angles and accuracy ranged from -10.7° to 4.2° where the presence of an athlete-specific bias was observed especially for the flexion angle. The improved joint drift correction reduced azimuth drift from over 25° to less than 5°. In conclusion, the system was valid for measuring 3D joint angles during alpine skiing and could be used outdoors. Errors were similar to the values reported in other studies for gait. The system may be well suited for within-athlete analysis but care should be taken for between-athlete analysis because of a possible athlete-specific joint angle bias.

## Introduction

Tracking of body segments and joints is traditionally performed with stereo-photogrammetric marker-based motion capture systems. Excluding errors from soft tissue artefacts (STA) such systems can measure three-dimensional (3D) positions and orientations of segments with an accuracy of <0.2 mm and <0.6°, respectively [1,2]. Joint orientations can be computed by calculating the relative orientation between two adjacent segments following ISB recommendations [3,4]. While such systems are well suited for in-lab measurements with relatively small capture volumes of a few cubic meters, they become unsuitable for larger volumes, such as often present in outdoor sports. For such sport applications inertial sensors have been proposed instead; e.g. to measure the kinematics of ski jumping [5], to estimate the instantaneous velocity for front-crawl swimming [6], to estimate spatio-temporal parameters in cross-country skiing [7], or to estimate temporal parameters during sprint running [8]. They are especially well suited for sports movement analysis because of their small size, possibility of being integrated into sports equipment or clothing, low dependence on environmental conditions (e.g. weather), and autonomy offering a pervasive monitoring.

However, inertial sensors cannot measure segment orientations directly. In order to obtain segment orientations with inertial sensors, several steps are required: 1) functional or anatomical calibration to align the sensor frame with the segment frame, 2) estimation of an initial segment orientation, and 3) mathematical procedure for tracking the change in segment orientation over time. Generally, this procedure is based on strap-down integration of angular velocity [9] combined with a drift reduction method [10–20]. Each step adds its own errors to the final segment orientation estimate: 1) misalignment from the anatomical or functional calibration, 2) inaccuracy of the initial segment orientation, and 3) lack of drift reduction.

In the past, different anatomical and functional calibrations were proposed mainly for gait analysis. Favre (2009) [21] proposed a functional calibration based on active hip ab/adduction and passive shank movements in the sagittal and frontal planes performed by the examiner while the subject was sitting on a chair. Repeatability (i.e. dispersion, defined as the spread of orientation differences in the calibration quaternions obtained with different movement repetitions) was <2.4° for thigh and shank segment orientations. Palermo (2014) [22] used two static postures (standing and lying on a bed) to functionally calibrate lower trunk and lower limb inertial sensors. Picerno (2008) [23] proposed an anatomical calibration method based on palpation of anatomical landmarks for measuring hip, knee, and ankle joint angles. These studies defined repeatability as the angle’s standard deviation between trials. Repeatability values <4° for the lower limb joint angles was reported except for the internal/external rotation where repeatability was up to 7.3°. The calibration movements proposed in these studies have been proposed for gait analysis in clinical settings where the time limitation constraint and context are totally different than for in-field sport applications. In sport situations, e.g. alpine skiing, calibration should take minimal time and should be performed without external equipment such as a chair or bed. In addition, calibration movements could involve more complex movements and benefit from the athlete’s high movement control abilities.

Although rarely specified, a wrong choice of initial segment orientation can noticeably affect the performance of the subsequent orientation tracking by adding orientation offsets. It is generally assumed that an initial posture is known [19] or can be measured [10]. Fusion schemes have also been proposed where wrong initial conditions have only minimal impact on orientation tracking; however, at the cost of having wrong orientation estimates during the first few seconds of tracking [18]. Movement constraints and hypotheses for initialization were rarely stated explicitly. For the subsequent orientation tracking, the above cited methods were able to reduce drift sufficiently and to obtain accurate and precise estimates of segment orientations and joint angles for gait analysis. However, as mentioned before, these algorithms were designed for gait, indoor measurements, relatively slow movements, and movements mostly constrained to the sagittal plane. It remains unknown whether the results could be generalized to faster movements and movements taking place out of the sagittal plane such as present in sports. For example, it could be expected that fast movements diminish the performance of such algorithms: for slow movements, measured acceleration mainly reflects Earth’s gravity. Sensor drift can be estimated by comparing measured gravity and true gravity [17]. In fast movements, measured acceleration also contains acceleration from the movement itself (linear and rotational), thus masking Earth’s gravity. Thus, this approach is not suited for drift correction during such movements [24]. Instead, the concept joint acceleration could be used: suppose acceleration is measured at known locations on two segments connected by a common joint. Based on rigid body kinematics, the accelerations can be translated to this joint. Any deviation between the translated accelerations from either segment is caused by measurement errors, for example induced by drift. This concept using the joint acceleration constraint was successfully exploited in the study of Fasel (2017) [20] and validated for the case of a single turn of alpine ski racing. Accuracy and precision of the outside leg’s knee flexion were 1.7° and 4.3°, respectively. However, the observed accuracy greatly varied between runs (standard deviation of 7.9°). Poorly performed calibration movements might be one plausible explanation for this observation. Accordingly, optimized calibration movements might help to improve the accuracy being achieved. Moreover, since the study was limited to flexion angles, it is still unclear how well the proposed joint drift reduction approach performs regarding the other two 3D angle components (i.e. ad/abduction and inter-external rotation angles).

Therefore, the aim of this study was (1) to propose and validate an improved functional calibration which is fast and usable in-field, and uses available sports equipment components (e.g. ski boots, poles) only; (2) to validate the 3D joint angles of the knee, hip, and trunk obtained by the use of this functional calibration for relatively long measurement durations (>30 seconds) in order to evaluate the impact of drift reduction.

## Methods

### Measurement protocol

**Fig 1:**
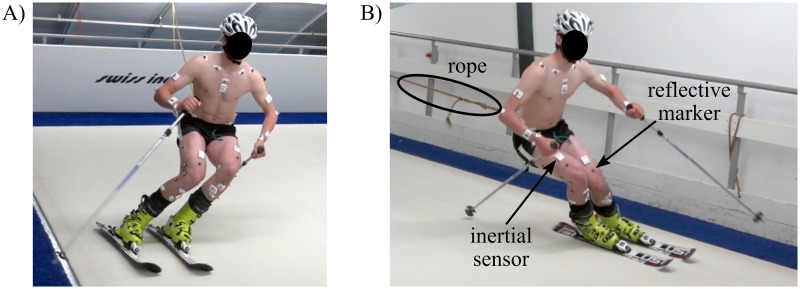
Carpet skiing. Illustration of skiing on the indoor skiing carpet for trial condition 110A, wide turns. A) left turn, B) right turn. The rope was connecting an external weight with the athlete’s belt for keeping him centred on the carpet. The inertial sensors can be identified as the small white boxes and the reflective markers as the small grey dots. The carpet surface was designed such that ski gliding friction is minimized.

**Fig 2:**
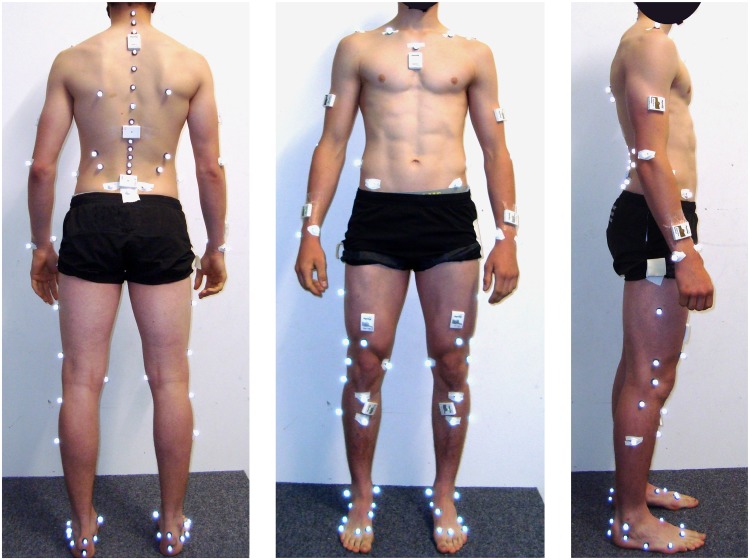
Sensor and marker placement. Sensor and marker placement from the back, front and side view. One additional inertial sensor was fixed to the athlete’s helmet (not shown).

Eleven male competitive alpine skiing athletes (20.9 years [15–30 years], 176.1 cm [164.5–185.0 cm], 74.0 kg [52.1–84.1 kg]) were enrolled in the study. The participating athletes were recruited in early 2016 in consultation with the local ski racing associations and the indoor skiing carpet operator. Inclusion criteria were male sex, a former history in competitive alpine ski racing and previous experience in indoor carpet skiing. Exclusion criterion was a recent not fully rehabilitated injury. All subjects invited, accepted and finally participated in the study. The study was approved by the EPFL Human Research Ethics Committee (Study Number: HREC 006–2016) and athletes gave written informed consent prior to the measurements. For the underage athletes additional written informed consent was obtained from their parents. The athlete depicted in Figs 1 and 2 in this manuscript has given written informed consent (as outlined in PLOS consent form) to publish his photographs. The measurement protocol consisted of skiing on a specially designed indoor skiing carpet (Maxxtracks Indoor Skislopes, The Netherlands) with dimensions 6 m × 11 m and 12° inclination (Fig 1). After warming up and familiarization, athletes skied a total of four trials at 21 km/h. Each trial lasted 120 seconds and was divided in two parts during which wide (entire carpet width) and narrow (half carpet width, marked with cones) turns were skied respectively. This within-trial protocol was applied to long radii turns (140 cm long skis with a sidecut radius of 11 m) and short radii turns (110 cm long skis with a sidecut radius of 8 m), for which two trials were performed each (Table 1). A custom made belt exerted a variable backwards force to ensure that the athlete remained in the central part of the carpet (Fig 1). Basic motion tasks (BMT, [25]) for the reference system where performed once at the very beginning. The calibration movements (FC1-FC5) for the wearable system were performed before each trial and after the last trial (Table 1).

**Table 1:** Overview of measurement protocol.

Trial code	Ski length	Speed	Turn types
BMT	-	-	-
FC1	-	-	Functional calibration 1
140A (test)	140 cm	21 km/h	45 sec wide / 45 sec narrow
FC2	-	-	Functional calibration 2
140B (retest)	140 cm	21 km/h	45 sec wide / 45 sec narrow
FC3	-	-	Functional calibration 3
110A (test)	110 cm	21 km/h	45 sec wide / 45 sec narrow
FC4	-	-	Functional calibration 4
110B (retest)	110 cm	21 km/h	45 sec wide / 45 sec narrow
FC5	-	-	Functional calibration 5

Order of tested skiing conditions, measured basic motion tasks (BMT) to calibrate the reference system and functional calibrations (FC1 –FC5) for the wearable system.

### Reference system

The reference system consisted of ten infrared cameras (Vicon Peak, UK) covering the volume spanned by the skiing carpet. Sampling frequency was set at 100Hz. Athletes were equipped with the IfB marker set [25–27] (Fig 2). Joint centres were determined functionally based on the data collected during the basic motion tasks. This setup allowed measuring 3D joint angles of ankle, knee, hip, and trunk [25] following the recommendations of Grood and Suntay [28]. Joint angles were set to zero during a barefoot standing trial. Then, the feet markers were put on the ski shoes and a static trial was used to define the functionally determined ankle joint centre with respect to the four shank markers (without the malleoli markers). The assumption was made that the foot was parallel to the sole of the ski boot. Therefore, ankle angels represent the angle between the shank segment and the rigid foot segment of the ski boot.

### Wearable system

Nine inertial sensors (Physilog 4, Gait Up, Switzerland) were attached with adhesive tape to the shanks, thighs, lower back (L5-L4 transition), sternum, upper back (T2-C7 transition), and head (Fig 1). Additional sensors not used in the present study were fixed slightly below T11 and to the upper limbs. The inertial sensors measured acceleration and angular velocity at 500 Hz. Accelerometer offset and sensitivity were corrected according to [29]. Gyroscope offset was estimated during the upright posture of the functional calibration. The wearable system was synchronized with the reference system by an electronic trigger.

#### Functional calibration

In this study it is assumed that the functional calibration is a procedure to estimate the calibration quaternion which rotates the sensor frame to their corresponding segment anatomic frame. It was achieved based on the following movements: squats, trunk rotation, hip ad/abduction, and upright standing. The movements have been optimized to be performed in a minimum time while wearing ski boots and on-snow, requiring no special equipment such as a scale or chair. Instructions on how to perform these movements and which movement was used to calibrate which sensor are provided in Table 2 and are further detailed on protocols.io under the DOI 10.17504/protocols.io.itrcem6. The matlab source code along with example data is provided on Code Ocean under the DOI 10.24433/CO.3f699198-4e77-4d51-8482-13d1b9ad93b8. Segment and joint coordinate systems were defined according to the ISB recommendations [3,4].

**Table 2:** Functional calibration movements.

Movement	Instruction	Calibrated segments	Calibrated axes
Squats	Slow squats with knee, hip, trunk, head flexion. Arms are parallel to leg, flex until fingers reach the ankles. Perform the movement three times.	Shank, thigh, lower back, upper back, sternum, head	Medio-lateral
Trunk rotations	Slow trunk rotations around the vertical axis with hips fixed. Arms hold a ski pole lying horizontally behind the neck. Head turns with the trunk. Perform the movement three times where rotation starts by looking to the right.	Lower back, upper back, sternum	Inferior-superior
Hip Ad/abductions	Slow hip ad/abductions of the right leg. Control balance using the ski poles. Right heel is positioned in-line with left toe. Keep knee straight through the entire movement. Perform the movement of slow hip abduction and adduction three times. Then perform the same for left leg.	Shank, thigh	Anterior-posterior
Upright	Stand upright with knees slightly flexed. Keep equal weight on both feet. Look straight to the front. Stand still for 10 seconds.	Shank, thigh, lower back, upper back, sternum, head, head	Inferior-superior

Description of the functional calibration movements used to align the sensor axes to the segment axes.

Since the movements were performed in ski boots they might not be executed properly, potentially leading to misalignment of the estimated anatomical frames. In order to counteract this problem, the segment’s functional axes and “zero” joint angles were approximated by combining different calibration movements and biomechanical constraints according to the following hypotheses. The trunk and head sensors were calibrated based on the following hypotheses: 1) squat movements occur around the medio-lateral axis, 2) trunk rotations are performed along the vertical axis, 3) during upright posture the trunk segment is perfectly vertical (i.e. no flexion and lateral bending). The thigh sensors were calibrated based on the following hypotheses: 1) squat movements occur around the medio-lateral axis, 2) orientation differences of measured lower back and thigh acceleration translated to the hip joint centre are minimal, where acceleration was translated according to Eqs 3 and 4. For the optimization procedure it was sufficient to fix the sensor-to-hip-joint-centre distance for the lower back sensor to (0.05 m, -0.10 m, 0.00 m) along the anterior-posterior, superior-inferior, lateral-medial anatomical axes. For the thigh it was sufficient to fix the sensor-to-hip-joint-centre distance at (-0.05 m, 0.30 m, 0.00 m). Shank sensors were calibrated according to the following hypotheses: 1) ad/abduction occurred around the anterior-posterior axis, 2) at the beginning of the ad/abduction movement the medio-lateral axis is perpendicular to gravity. Finally, the lower limb calibration was optimized according to the following hypotheses: 1) average knee flexion during the hip ad/abduction is zero, 2) medio-lateral axis of shank and thigh is perpendicular to gravity during upright standing, 3) left and right shanks and thighs have the same segment orientation at the beginning of the squat movement.

#### Estimating initial orientation

The segment orientations were estimated using the strap-down integration and joint drift correction presented in [20]. The global frame was defined as follows: the Y-axis was aligned with gravity, pointing upwards. X-axis was perpendicular to gravity and pointing forwards in the direction of the fall-line. The Z-axis was the cross-product between the X- and Y-axis, pointing to the right. For determining the initial conditions of the strap-down integration, it was assumed that all trunk and lower limb segments had the same azimuth (i.e. were heading the same direction). The segments’ inclinations were determined using gravity.

#### Improved drift correction

Let’s consider ***a***_***distal***_(***t***) and ***a***_***proximal***_(***t***) as the accelerations measured by the distal and proximal sensors placed at a certain distance ***r***_***d***_ and ***r***_***p***_ from the connecting joint, and ${\tilde{a}}_{distal}^{G}\left(t\right)$ and ${\tilde{a}}_{proximal}^{G}\left(t\right)$ as the distal and proximal accelerations translated to the connecting joint (Eqs 1–4). As proposed by [20], theoretically there should not be any difference in orientation between ${\tilde{a}}_{distal}^{G}\left(t\right)$ and ${\tilde{a}}_{proximal}^{G}\left(t\right)$. Therefore, any orientation difference should express only error. In [20] this error was considered as drift and was expressed by the quaternion ***δ***(***t***) (Eqs 5–7).

$${\tilde{a}}_{distal}^{G}\left(t\right)=R_{distal}^{G}\left(t\right)\;{\tilde{a}}_{distal}\left(t\right)$$ (1)

$${\tilde{a}}_{proximal}^{G}\left(t\right)=R_{proximal}^{G}\left(t\right)\;{\tilde{a}}_{proximal}\left(t\right)$$ (2)

$${\tilde{a}}_{distal}\left(t\right)=\;a_{distal}\left(t\right)+\;{\dot{\omega }}_{distal}\left(t\right)\times r_{d}+\;\omega_{distal}\left(t\right)\;\times \left(\omega_{distal}\left(t\right)\times r_{d}\right)$$ (3)

$${\tilde{a}}_{proximal}\left(t\right)=\;a_{proximal}\left(t\right)+\;{\dot{\omega }}_{proximal}\left(t\right)\times r_{p}+\;\omega_{proximal}\left(t\right)\;\times \;\left(\omega_{proximal}\left(t\right)\times r_{p}\right)$$ (4)

where $R_{distal}^{G}\left(t\right)$ and $R_{proximal}^{G}\left(t\right)$ are the drift-affected orientations at time *t* of the distal and proximal segment estimated by the strap-down integration expressed in the global frame and ***ω***_*distal*_(*t*) and ***ω***_*proximal*_(*t*) are the angular velocities of the distal and proximal segments.

$$\delta \left(t\right)=\;\left[cos\;\left(\frac{\beta \left(t\right)}{2}\right),\text{ sin }\left(\frac{\beta \left(t\right)}{2}\right)\cdot U\left(t\right)\right]$$ (5)

where *β*(*t*) and *U*(*t*) are the axis-angle representation of quaternion *δ*(*t*) (Eqs 6 and 7):

$$\beta \left(t\right)=\text{acos }\left(\frac{{\tilde{a}}_{distal}^{G}\left(t\right)\cdot {\tilde{a}}_{proximal}^{G}\left(t\right)}{\left|{\tilde{a}}_{distal}^{G}\left(t\right)\left|\cdot \right|{\tilde{a}}_{proximal}^{G}\left(t\right)\right|}\right)$$ (6)

$$U\left(t\right)=\;\frac{{\tilde{a}}_{distal}^{G}\left(t\right)\times {\tilde{a}}_{proximal}^{G}\left(t\right)}{\left|{\tilde{a}}_{distal}^{G}\left(t\right)\times {\tilde{a}}_{proximal}^{G}\left(t\right)\right|}$$ (7)

Drift was estimated for all time instants *t* satisfying Eqs 8–10, by considering high signal to noise ratio:

$$\left|\left|{\tilde{a}}_{distal}^{G}\left(t\right)\right|-\left|{\tilde{a}}_{proximal}^{G}\left(t\right)\right|\right|<th_{min}$$ (8)

$$\left|{\tilde{a}}_{distal}^{G}\left(t\right)\right|>th_{max}$$ (9)

$$\left|{\tilde{a}}_{proximal}^{G}\left(t\right)\right|>th_{max}$$ (10)

In our previous study, *th*_*min*_ and *th*_*max*_ were selected to 2.5 m/s^2^ and 8 m/s^2^ to include only samples with high signal to noise ratio. However, these thresholds were good for on-snow skiing with relatively high accelerations. Since skiing speed for indoor carpet skiing was substantially lower, *th*_*max*_ was fixed to 6 m/s^2^ and the constraint on *th*_*min*_ was adapted to include all samples with less than 20% acceleration magnitude difference (Eq 11):

$$\frac{\left|\left|{\tilde{a}}_{distal}^{G}\left(t\right)\right|-\left|{\tilde{a}}_{proximal}^{G}\left(t\right)\right|\right|}{0.5*\left(\left|{\tilde{a}}_{distal}^{G}\left(t\right)\right|+\left|{\tilde{a}}_{proximal}^{G}\left(t\right)\right|\right)}<0.2$$ (11)

**Fig 3:**
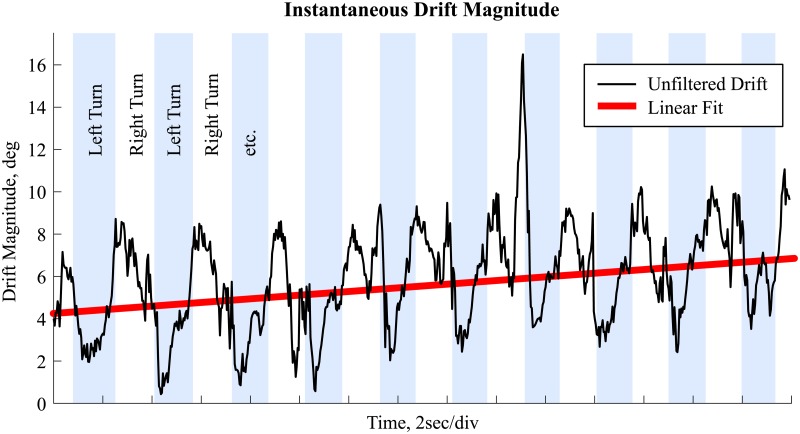
Drift magnitude. Estimated drift magnitude for the left thigh for 30 seconds of a typical trial showing the existence of noise due to kinematic components of the movement. Left turns are marked in light blue. The red line shows a linear fit to the drift magnitude for illustration purpose only.

Not all orientation misalignments between ${\tilde{\;a}}_{distal}^{G}\left(t\right)$ and ${\tilde{a}}_{proximal}^{G}\left(t\right)$ could be explained by drift. In addition to drift, other estimation error sources might be present: inaccurately estimated ***r***_*d*_ and ***r***_*p*_ or different kinematics for the distal and proximal segments (e.g. different soft tissue artefact in the proximal segment compared to the distal segment). As can be noticed in Fig 3, in addition to a potentially linear drift, the instantaneous drift magnitude *δ*(*t*) is correlated to the changing knee angle between left and right turns. Therefore, to minimize the movement’s influence on the estimated drift, *δ*(*t*) should be averaged over at least one movement cycle. For this study, we chose to average over two movement cycles. A movement cycle was determined to include a left and a right turn. It was assumed that a movement cycle starts at a local maximum of the segment’s angular velocity in the global frame’s X-axis.

For estimating the drift, axes with larger accelerations are weighed more. Due to Earth’s gravity there is always considerable acceleration along the vertical axis. Thus, the proposed joint drift method may miss drift along the azimuth axis since accelerations in the horizontal plane are too small compared to the acceleration along the vertical axis. Therefore, joint drift was corrected a second time by setting all acceleration along the vertical axis in the global frame to zero (Eqs 12 and 13).

$${\hat{a}}_{distal}^{G}\left(t\right)={\left[{\tilde{a}}_{distal,\;X}^{G}\left(t\right),\;0,\;{\tilde{a}}_{distal,Z}^{G}\left(t\right)\right]}^{T}$$ (12)

$${\hat{a}}_{proximal}^{G}\left(t\right)={\left[{\tilde{a}}_{proximal,\;X}^{G}\left(t\right),\;0,\;{\tilde{a}}_{proximal,Z}^{G}\left(t\right)\right]}^{T}$$ (13)

Since the vertical axis has been set to zero, the threshold *th*_*max*_ (Eqs 9 and 10) for valid drift estimation samples had to be adapted and was set to *th*_*min*_ = 0.6 *m*/*s*^2^ where a trade-off between strict conditions and enough available valid samples had to be found. The drift was averaged over the same time windows as for the initial estimation.

#### Computing the joint angles

The 3D joint angles of the knee and hip were computed following the ISB recommendations [3] and Grood and Suntay [28]. Knee angles ***α***_**[left/right] knee**_**(*t*)** were computed based on the shank and thigh orientations. Hip angles ***α***_**[left/right] hip**_**(*t*)** were computed based on the thigh and lower back orientations. The 3D joint angles for the trunk were computed using a slightly adapted version of Grood and Suntay as done in earlier studies [30,31]. The trunk angles were computed in two ways: ***α***_**l.back−stern**_**(*t*)** used the lower back and sternum orientation, as in [20,30]. ***α***_**l.back−u.back**_**(*t*)** used the lower back and upper back orientation.

### Validation

#### Functional calibration

The proposed functional calibration procedure was validated based on the repeatability of the calibration quaternions (i.e. influence of the movements on the calibration quaternion) and on the repeatability of the 3D knee, hip, and trunk angles (i.e. error propagation from calibration quaternion to joint angles). Both quantities were defined as proposed by [21] where the repeatability of the calibration quaternion was defined as the dispersion ***χ*** of the five calibration quaternions ***q***_**A,F**_ (for each athlete ***A*** and each functional calibration ***F***) around their mean ***q***_***A***_ for all athletes (Eqs 14 and 15).

$$\chi =\sqrt{\frac{1}{A*F-1}\;\sum_{A,\;F}\Delta_{A,F}^{2}}$$ (14)

$$\Delta_{A,F}=2\text{ cos }\left({\left|q_{A}\;⊗\;q_{A,F}^{-1}\right|}_{real}\right)$$ (15)

where Δ_*A*,*F*_ corresponds to the orientation angle difference between *q*_*A*_ and *q*_*A*,*F*_. *A* denotes the athletes {1, …, 10}, *F* the functional calibrations {1, …, 5}, and ⊗ the quaternion multiplication.

The repeatability of the 3D joint angles was obtained by computing first an average joint angle ${\bar{\alpha }}_{A,J}\left(t\right)$ for each angle *J* and athlete *A* based the mean of the five functional calibrations applied to the same trial (Eq 16).

$${\bar{\alpha }}_{A,J}\left(t\right)=\frac{1}{5}\overset{5}{\underset{F=1}{\sum }}\alpha_{A,J,F}\left(t\right)$$ (16)

Trial 110A has been chosen for this purpose, as the shorter skis allow narrower and thus more dynamic turns. Then, the difference between the five angles *α*_*A*,*J*,*F*_(*t*) and ${\bar{\alpha }}_{A,J}\left(t\right)$ was computed for each athlete and their mean ${\text{Λ}}_{A,J,F}^{mean}$ and standard deviation ${\text{Λ}}_{A,J,F}^{std}$ was computed over time. Next, the mean absolute deviation of ${\text{Λ}}_{A,J,F}^{mean}$ and the mean of ${\text{Λ}}_{A,J,F}^{std}$ was computed by averaging over the five functional calibrations for each athlete. Finally, these values were averaged over all athletes to obtain the offset ${\text{Λ}}_{J}^{mean}$ and precision ${\text{Λ}}_{J}^{std}$ for each joint angle. The coefficient of multiple correlation *CMC*_*J*,*A*_ was computed between *α*_*A*,*J*,*F*_(*t*) of the five functional calibrations and then averaged over all athletes to obtain *CMC*_*J*_ [21,32].

#### Joint angles

The 3D joint angles computed with the wearable system were down-sampled to 100 Hz to match the sampling frequency of the reference system. For each of the four trials per athlete the functional calibration immediately preceding the trial was taken. The joint angle error ***ϵ***_**A,T,J**_**(*t*)** was defined as the sample-by-sample difference between the wearable and the reference system for trial ***T*** (Eq 17).

$$\epsilon_{A,T,J}\left(t\right)=\alpha_{A,T,J}^{wearable}\left(t\right)-\alpha_{A,T,J}^{reference}\left(t\right)$$ (17)

Per-Trial accuracy and precision were then defined as the mean *μ*_*A*,*T*,*J*_ and standard deviation *σ*_*A*,*T*,*J*_ of *ϵ*_*A*,*T*,*J*_(*t*) over time. The relationship between the joint angles obtained with the wearable and reference systems was assessed by Pearson’s correlation coefficient *c*_*A*,*T*,*J*_. Overall accuracy and precision were then defined as the average and standard deviation of *μ*_*A*,*T*,*J*_, respectively *σ*_*A*,*T*,*J*_, computed over all trials and athletes. Overall correlation was obtained the same way.

## Results

Functional calibration and joint angle validity could be assessed for all 11 athletes and trials, resulting in a total of 44 trials. Results for left and right side were similar. Thus, in the following only the results for the left side are presented. Similarly, no differences between upper trunk orientation computed from the sternum or upper trunk sensors were found. The results for trunk segment and joint orientation are only shown with respect to the lower trunk–sternum sensors. S1–S3 Tables provide an exhaustive presentation of the results for both left and right sides and for the trunk angles computed based on the upper trunk sensor.

### Validation functional calibration

Dispersion (*χ*) of the calibration quaternion ranged from 5.5° for the shank to 1.6° for the sternum (Table 3). Joint angle repeatability offset (${\text{Λ}}_{J}^{mean}$) was <2.7° for all angles. Generally, offsets for the flexion axis were 1° larger than the other axes. Repeatability standard deviation (${\text{Λ}}_{J}^{std}$) ranged between 0.5° and 1.5°. Average CMC was >0.87 for the lower limbs and >0.81 for the neck but lower for the trunk with a minimum CMC of 0.5 for trunk flexion (Table 4).

**Table 3:** Dispersion of the calibration quaternions.

Segment	Dispersion *χ*
Left Shank	5.50°
Left Thigh	2.94°
Lower back	4.11°
Sternum	1.57°
Head	3.13°

**Table 4:** Joint angle repeatability.

		Repeatability offset	Repeatability standard deviation	CMC
Left Knee	Flexion, deg:	2.0 (0.7)	1.4 (0.6)	0.934 (0.021)
	Abduction, deg:	1.0 (0.4)	0.5 (0.2)	0.941 (0.055)
	Rotation, deg:	0.8 (0.3)	0.7 (0.2)	0.932 (0.059)
Left Hip	Flexion, deg:	2.7 (1.1)	0.5 (0.2)	0.957 (0.031)
	Abduction, deg:	1.2 (1.0)	0.7 (0.2)	0.866 (0.287)
	Rotation, deg:	1.3 (0.9)	0.7 (0.3)	0.970 (0.043)
Trunk	Flexion, deg:	2.1 (1.2)	0.3 (0.3)	0.490 (0.335)
	Abduction, deg:	1.5 (1.4)	0.3 (0.3)	0.741 (0.402)
	Rotation, deg:	0.7 (0.8)	0.4 (0.3)	0.883 (0.271)
Neck	Flexion, deg:	2.2 (1.1)	0.4 (0.3)	0.835 (0.162)
	Abduction, deg:	1.1 (1.4)	0.6 (0.4)	0.864 (0.229)
	Rotation, deg:	1.8 (2.3)	0.8 (0.6)	0.808 (0.301)

The table reports mean values (standard deviation) for the calibration repeatability of all eleven athletes.

### Validation joint angles

**Fig 4:**
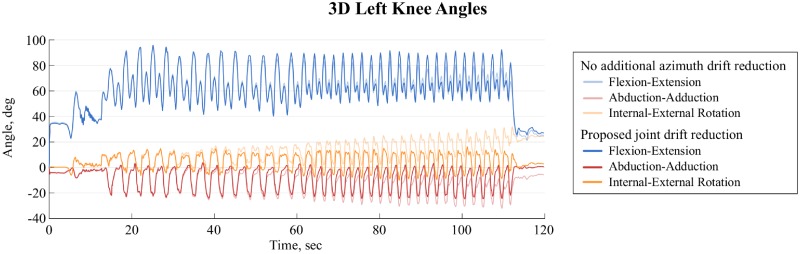
3D knee angles with and without azimuth drift correction. Comparison of the 3D knee angles for the joint drift reduction without the proposed azimuth drift correction (light colours) and with the additional azimuth drift correction (dark colours).

**Fig 5:**
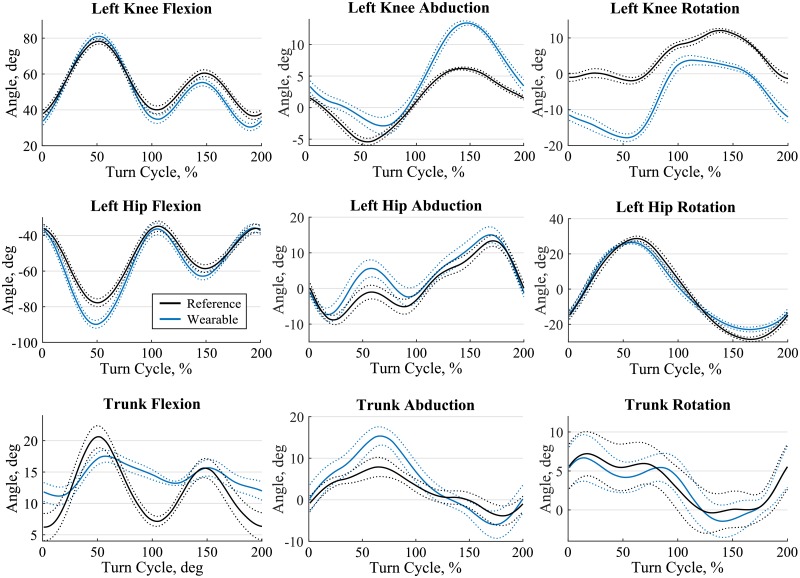
Typical joint angles. Time normalized joint angles for the knee, hip and trunk of a typical athlete for 13 wide double turns (left and right turn) of trial 110B. The first 100% of the turn cycle is a left turn where the left leg is the inside leg. The second 100% of the turn cycle is a right turn where the left leg is the outside leg. Solid line is the average and dotted the standard deviation. Black is the reference system and blue the wearable system.

Reference angle minima and maxima were largest for knee and hip flexion with 36.3°– 74.7° and -67.2 –-24.8°, respectively. They were smallest for trunk abduction with ±6.3° (Table 5). Accuracy ranged from -10.7° for the left hip flexion to 4.2° for the left knee abduction. Precision ranged from 2.2° for the trunk flexion up to 4.9° for the left hip internal rotation (Table 5). Correlation between the wearable and reference system was above 0.9 except for the left knee internal/external rotation and for all three trunk angles (Table 5). The adapted joint drift correction proposed in this study allowed to reduce the azimuth drift. For a typical trial 3D knee joint angles obtained with and without the proposed azimuth drift correction are compared in Fig 4. Azimuth drift correction allowed to reduce azimuth drift and also decreased axis cross-talk for the flexion and ad/abduction angles. For illustration purposes, joint angles for a typical trial of condition 110B were segmented into double turns (left and right turn), time-normalized and averaged for thirteen wide turns (Fig 5).

**Table 5:** 3D joint angles range of motion and errors.

		Reference		Wearable		Error		
		*Min*.	*Max*.	*Min*.	*Max*.	*Accuracy*	*Precision*	*Correlation*
Left Knee	Flexion, deg	36.3 (5.6)	74.7 (8.4)	33.9 (6.5)	77.1(12.2)	-0.1 (7.4)	3.4 (1.4)	0.955 (0.043)
	Abduction, deg	-11.6 (4.1)	2.3 (2.9)	-11.5 (6.3)	11.9 (4.9)	4.2 (5.5)	3.6 (0.9)	0.919 (0.094)
	Rotation, deg	-9.4 (3.7)	10.2 (3.4)	-11.4 (4.4)	11.2 (4.8)	0.0 (4.4)	3.8 (1.2)	0.781 (0.172)
Left Hip	Flexion, deg	-67.2 (11.0)	-24.8 (7.9)	-82.7 (10.5)	-32.6 (8.8)	-10.7 (4.3)	3.6 (1.3)	0.974 (0.016)
	Abduction, deg	-10.5 (5.6)	16.5 (6.2)	-14.7 (5.7)	14.0 (7.6)	-3.3 (4.1)	3.1 (1.4)	0.896 (0.135)
	Rotation, deg	-30.5 (5.9)	23.6 (5.7)	-24.0 (5.4)	21.1 (7.0)	0.5 (4.8)	4.9 (1.5)	0.977 (0.013)
Trunk	Flexion, deg	3.7 (5.6)	16.6 (5.6)	5.8 (5.5)	16.6 (4.9)	1.1 (6.4)	2.2 (0.9)	0.711 (0.208)
	Abduction, deg	-6.3 (3.2)	6.3 (3.8)	-8.7 (3.7)	8.5 (3.6)	0.1 (3.6)	2.6 (0.9)	0.790 (0.199)
	Rotation, deg	-6.7 (4.4)	7.0 (3.5)	-10.2 (7.0)	9.4 (4.0)	-0.6 (2.5)	3.6 (1.5)	0.669 (0.309)

Reference and wearable minimum and maximum angles and accuracy (error mean), precision (error standard deviation), and correlation. Values are given as mean (standard deviation) of all trials.

## Discussion

In this study a new functional calibration that can easily be used in-field was proposed and validated. The calibration movements were designed such they could be performed wearing ski boots and using ski poles. In addition, the previously presented joint drift correction method [20] was improved (Fig 4). 3D joint angles of the knee, hip and trunk estimated with the inertial sensors (wearable system) were validated against reference angles obtained with a marker-based stereo-photogrammetric system during indoor carpet skiing.

### Functional calibration

Functional calibration movements proposed in the past required either active or passive movements of the lower limbs [21] or standing and lying postures [23]. Since these movements were not dedicated to outdoor movements and cannot be performed reliably by athletes wearing ski boots, new and adapted functional calibration movements have been proposed. The main difficulty comes from aligning the inertial sensors to the body segments in the sagittal plane. The ski boots imposed an ankle flexion of approximately 17° in standing posture, making impossible the acquisition of a neutral pose required to initialize joint angles to 0° [21,33,34]. Thus, in the proposed scenario, the “zero” joint angle was approximated by combining the different calibration movements and biomechanical constraints. In addition to that, the proposed approach was sufficiently repeatable: joint angle offsets between different repetitions of functional calibrations were below 2.7° and their impact on joint angle precision was below 1.4° (Table 4). CMC for trunk angles was low, probably due to the small angle ranges (Table 5). The results are comparable to previous studies which also reported repeatability ranging between 2° and 4° for most joint angles [21–23]. Despite this high repeatability, a comparatively high standard deviation of joint flexion angle offsets (up to 7.4° for the knee, Table 5) was observed. Post-hoc one-way ANOVA for the knee flexion offset showed that 86.5% of its total variance was explained by the between-athlete variance and only 13.5% was explained by the within-athlete variance. Thus, the functional calibration provided highly repeatable results within athletes, but not between athletes. The computed joint angles contained an athlete-specific bias. While this could easily be corrected with a neutral posture without ski boots, further work may be required to remove the athlete-specific bias when wearing ski boots.

### Joint angles

The 3D joint angles estimated with the wearable system showed a good agreement to the reference system (precision of 2.2°– 4.9°, correlation >0.9 for most joint angles). In a similar validation study focusing on sports movements and using a Kalman filter to estimate orientations [16] reported knee flexion root mean square error (RMSE) between 7.0° for walking to 10.2° for running with correlation values >0.95. However, they partly removed systematic offsets between wearable and reference system for each trial prior to computing the RMSE, making a comparison to our results difficult. We chose not to align the anatomical and functional frames of both systems, since we wanted to assess how well the proposed functional calibration is able to approximate the joint kinematics in the anatomical frame. Compared to [21] accuracy of the proposed system (mean absolute difference (standard deviation) of 5.7° (4.7°), 6.0° (3.4°), 3.2° (3.0°) for left knee flexion, abduction, and rotation, respectively) was better, but precision was worse. Improved accuracy could be explained by the different functional calibration procedure. The worse precision could be explained by the higher joint range of motion and movement dynamic, and, therefore, a different (potentially increased) amount of soft tissue artefact [35]. However, even though marker setup was chosen such as to minimize influence of soft tissue artefacts and joint angle estimation errors due to small errors in marker placement [25], knee ad/abduction and internal/external angles should be interpreted with care; both for the reference system and for the wearable system. Precision was best for the trunk angles, however the correlation between the wearable and reference system was below 0.8. On the one hand, these small correlation values could originate in the small range of motion of only 15°– 20° for all axes. On the other hand, the different definition of trunk angles between the two systems could also explain the reduced correlation: while for the reference system the trunk angles were defined as the orientation difference between the pelvis and cervical spine segments, for the wearable system the trunk angles were defined as the orientation difference between the lower back and sternum. This different definition had to be used, as due to equipment- and movement-related restrictions a direct fixation of one or multiple inertial on the pelvis was not feasible, and an alternative solution (i.e. fixation on the sacrum) was indispensable. Since precision (but not accuracy) of all axes was good, the angle curves could be well suited for comparing differences in shape, but not absolute values. For example, the system would be suitable to detect angle pattern differences caused by a change of condition, such as the difference between different turn techniques or equipment used.

In this study, the long acquisition duration with both the reference and the wearable system allowed to validate the drift reduction algorithm for periods of up to two minutes. The improved joint drift reduction did allow a better drift reduction along the vertical axis (azimuth drift). The azimuth drift reduction did not only improve the joint’s internal-external angles, but also reduced axis-cross talk, improving the angles along all three axes (Fig 4). Moreover, since only acceleration and angular velocity were used, the system was independent from magnetic distortions as present indoors due to metallic structures. Therefore, in contrast to other drift reduction methods using magnetometer measurements, the system would be an ideal choice for indoor measurements on the skiing carpet and could also be used for other sports such as treadmill cross-country skiing.

### Methodological considerations

A limitation of the current study might have been the limited speed when skiing on the indoor carpet (21 km/h). As a consequence, joint accelerations were smaller and the thresholds for including valid samples for joint drift correction that were proposed in [20] had to be adapted. Since the proposed thresholds are dependent on the measured acceleration, they could also be used for on-snow measurements, where higher accelerations and joint ranges of motion are present. First, higher accelerations are expected to allow a more reliable estimation of joint drift since the relative impact of small errors (e.g. originating form sensor noise) is lower. Second, higher ranges of motion could increase the robustness of the joint drift estimation, since potential estimation errors at one particular joint orientation (e.g. 45° knee flexion) could be compensated with more estimates from other joint orientations (e.g. 90° knee flexion). Finally, the aforementioned conclusion is further supported by the findings of a recent study assessing the system’s accuracy and precision during outdoor skiing, in which for knee and hip flexion angles similar error magnitudes were observed [20]. Potential joint angle errors for on-snow measurements might be higher due to the ski chattering-induced vibration noise from the ski-snow interaction. This noise might reduce the observed systems precision by a few degrees but should be still smaller than the precision of 6° reported for the hip flexion in [20].

## Conclusion

An optimized functional calibration movement was proposed and validated. The wearable system was able to estimate the 3D joint angles for hip and trunk, as well as the knee flexion angle. The knee ad/abduction and internal/external rotation should be interpreted with care as the estimated angles may include axis-cross talk and soft tissue artefacts. The accuracy might not be sufficient for absolute angle comparisons across different athletes. However, the system should be sufficiently sensitive for within-athlete comparisons assessing the influence of certain conditions or interventions on joint kinematics. Further investigation should be targeted on reducing soft tissue artefacts of the thigh. In the context of coaching, the system could be used to provide athletes precise and objective feedbacks on their movement patterns and to improve their techniques. Moreover, knowing the joint ranges of motion and joint movement speeds, strength and conditioning trainings could be optimized and personalized.

## Supporting information

S1 Table Dispersion of the calibration quaternions. Average dispersion of the calibration quaternions around their mean.
(DOCX)

S2 Table Joint angle repeatability. The table reports mean values (standard deviation) for the calibration repeatability of all eleven athletes.
(DOCX)

S3 Table 3D joint angles range of motion and errors. Reference and wearable minimum and maximum angles and accuracy (error mean), precision (error standard deviation), and correlation. Values are given as mean (standard deviation) of all trials.
(DOCX)
