# Supplementary material for: Validation of functional calibration and strap-down joint drift correction for computing 3D joint angles of knee, hip, and trunk in alpine skiing
Source: PLoS One. 2017 Jul 26;12(7):e0181446. doi: 10.1371/journal.pone.0181446 (PMC5528837; doi:10.1371/journal.pone.0181446)
Supplement: S1 Table — Average dispersion of the calibration quaternions around their mean. (DOCX) [file pone.0181446.s001.docx]

| **Segment** | **Dispersion** $\boldsymbol{\chi}$ |
| --- | --- |
| Left Shank | 5.50° |
| Right Shank | 4.43° |
| Left Thigh | 2.94° |
| Right Thigh | 3.08° |
| Lower back | 4.11° |
| Upper back | 2.85° |
| Sternum | 1.57° |
| Head | 3.13° |
