# Supplementary material for: Validation of functional calibration and strap-down joint drift correction for computing 3D joint angles of knee, hip, and trunk in alpine skiing
Source: PLoS One. 2017 Jul 26;12(7):e0181446. doi: 10.1371/journal.pone.0181446 (PMC5528837; doi:10.1371/journal.pone.0181446)
Supplement: S2 Table — The table reports mean values (standard deviation) for the calibration repeatability of all eleven athletes. (DOCX) [file pone.0181446.s002.docx]

|  |  | **Repeatability Offset** | **Repeatability Standard Deviation** | **CMC** |
| --- | --- | --- | --- | --- |
| Left Knee | Flexion, deg: | 2.0 (0.7) | 1.4 (0.6) | 0.934 (0.021) |
|  | Abduction, deg: | 1.0 (0.4) | 0.5 (0.2) | 0.941 (0.055) |
|  | Rotation, deg: | 0.8 (0.3) | 0.7 (0.2) | 0.932 (0.059) |
| Right Knee | Flexion, deg: | 1.9 (0.5) | 1.3 (0.4) | 0.944 (0.032) |
|  | Abduction, deg: | 1.2 (0.5) | 0.6 (0.2) | 0.895 (0.113) |
|  | Rotation, deg: | 1.2 (0.6) | 0.7 (0.3) | 0.846 (0.149) |
| Left Hip | Flexion, deg: | 2.7 (1.1) | 0.5 (0.2) | 0.957 (0.031) |
|  | Abduction, deg: | 1.2 (1.0) | 0.7 (0.2) | 0.866 (0.287) |
|  | Rotation, deg: | 1.3 (0.9) | 0.7 (0.3) | 0.970 (0.043) |
| Right Hip | Flexion, deg: | 2.7 (1.0) | 0.5 (0.3) | 0.958 (0.034) |
|  | Abduction, deg: | 1.2 (0.7) | 0.8 (0.2) | 0.870 (0.262) |
|  | Rotation, deg: | 1.4 (1.0) | 0.7 (0.3) | 0.966 (0.040) |
| Trunk *(lower trunk-sternum)* | Flexion, deg: | 2.1 (1.2) | 0.3 (0.3) | 0.490 (0.335) |
|  | Abduction, deg: | 1.5 (1.4) | 0.3 (0.3) | 0.741 (0.402) |
|  | Rotation, deg: | 0.7 (0.8) | 0.4 (0.3) | 0.883 (0.271) |
| Trunk *(lower trunk – upper trunk)* | Flexion, deg: | 2.2 (1.3) | 0.4 (0.3) | 0.519 (0.357) |
|  | Abduction, deg: | 1.6 (1.3) | 0.4 (0.3) | 0.715 (0.370) |
|  | Rotation, deg: | 1.0 (0.8) | 0.7 (0.6) | 0.840 (0.302) |
| Neck | Flexion, deg: | 2.2 (1.1) | 0.4 (0.3) | 0.835 (0.162) |
|  | Abduction, deg: | 1.1 (1.4) | 0.6 (0.4) | 0.864 (0.229) |
|  | Rotation, deg: | 1.8 (2.3) | 0.8 (0.6) | 0.808 (0.301) |

The table reports mean values (standard deviation) for the calibration repeatability of all eleven athletes.
