# Supplementary material for: Validation of functional calibration and strap-down joint drift correction for computing 3D joint angles of knee, hip, and trunk in alpine skiing
Source: PLoS One. 2017 Jul 26;12(7):e0181446. doi: 10.1371/journal.pone.0181446 (PMC5528837; doi:10.1371/journal.pone.0181446)
Supplement: S3 Table — Reference and wearable minimum and maximum angles and accuracy (error mean), precision (error standard deviation), and correlation. Values are given as mean (standard deviation) of all trials. (DOCX) [file pone.0181446.s003.docx]

|  |  | **Reference** | | **Wearable** | | **Error** | | |
| --- | --- | --- | --- | --- | --- | --- | --- | --- |
|  |  | *Min.* | *Max.* | *Min.* | *Max.* | *Accuracy* | *Precision* | *Correlation* |
| Left Knee | Flexion, deg | 36.3  (5.6) | 74.7  (8.4) | 33.9  (6.5) | 77.1 (12.2) | -0.1  (7.4) | 3.4  (1.4) | 0.955 (0.043) |
|  | Abduction, deg | -11.6  (4.1) | 2.3  (2.9) | -11.5  (6.3) | 11.9  (4.9) | 4.2  (5.5) | 3.6  (0.9) | 0.919 (0.094) |
|  | Rotation, deg | -9.4  (3.7) | 10.2  (3.4) | -11.4  (4.4) | 11.2  (4.8) | 0.0  (4.4) | 3.8  (1.2) | 0.781 (0.172) |
| Right Knee | Flexion, deg | 36.3  (5.7) | 71.1  (6.8) | 32.4  (6.4) | 76.9  (9.6) | -1.3  (8.0) | 4.1  (2.7) | 0.926  (0.131) |
|  | Abduction, deg | -10.8  (2.8) | 2.9  (2.5) | -10.6  (6.5) | 13.0  (4.0) | 4.8  (3.7) | 3.8  (1.2) | 0.922  (0.085) |
|  | Rotation, deg | -8.6  (3.7) | 8.1  (4.9) | -9.8  (6.2) | 10.8  (5.8) | 0.8  (5.7) | 3.4  (1.2) | 0.796  (0.154) |
| Left Hip | Flexion, deg | -67.2 (11.0) | -24.8  (7.9) | -82.7 (10.5) | -32.6  (8.8) | -10.7 (4.3) | 3.6  (1.3) | 0.974 (0.016) |
|  | Abduction, deg | -10.5  (5.6) | 16.5  (6.2) | -14.7  (5.7) | 14.0  (7.6) | -3.3  (4.1) | 3.1  (1.4) | 0.896 (0.135) |
|  | Rotation, deg | -30.5  (5.9) | 23.6  (5.7) | -24.0  (5.4) | 21.1  (7.0) | 0.5  (4.8) | 4.9  (1.5) | 0.977 (0.013) |
| Right Hip | Flexion, deg | -68.1  (10.5) | -25.8  (8.1) | -83.1  (9.6) | -31.0  (8.4) | -9.1  (4.9) | 4.1  (1.2) | 0.966  (0.026) |
|  | Abduction, deg | -11.6  (4.3) | 15.5  (7.1) | -13.1  (5.4) | 14.6  (8.1) | -1.4  (3.5) | 3.2  (1.4) | 0.882  (0.173) |
|  | Rotation, deg | -22.4  (5.5) | 31.7  (4.9) | -20.7  (8.5) | 22.9  (5.1) | -2.8  (3.6) | 5.2  (2.1) | 0.980  (0.011) |
| Trunk *(lower trunk-sternum)* | Flexion, deg | 3.7  (5.6) | 16.6  (5.6) | 5.8  (5.5) | 16.6  (4.9) | 1.1  (6.4) | 2.2  (0.9) | 0.711 (0.208) |
|  | Abduction, deg | -6.3  (3.2) | 6.3  (3.8) | -8.7  (3.7) | 8.5  (3.6) | 0.1  (3.6) | 2.6  (0.9) | 0.790 (0.199) |
|  | Rotation, deg | -6.7  (4.4) | 7.0  (3.5) | -10.2  (7.0) | 9.4  (4.0) | -0.6  (2.5) | 3.6  (1.5) | 0.669 (0.309) |
| Trunk *(lower trunk – upper trunk)* | Flexion, deg | 3.7  (5.6) | 16.6  (5.6) | 6.6  (6.2) | 17.9  (5.9) | 2.4  (6.0) | 2.0  (0.8) | 0.754  (0.164) |
|  | Abduction, deg | -6.3  (3.2) | 6.3  (3.8) | -8.7  (4.1) | 8.5  (4.3) | 0.1  (3.7) | 2.7  (1.0) | 0.762  (0.254) |
|  | Rotation, deg | -6.7  (4.4) | 7.0  (3.5) | -10.4  (6.5) | 11.4  (4.0) | 0.3  (4.0) | 4.2  (1.4) | 0.635  (0.369) |

Reference and wearable minimum and maximum angles and accuracy (error mean), precision (error standard deviation), and correlation. Values are given as mean (standard deviation) of all trials.
